# Screening for DAX1/EWS‐FLI1 functional inhibitors identified dihydroorotate dehydrogenase as a therapeutic target for Ewing's sarcoma

**DOI:** 10.1002/cam4.5741

**Published:** 2023-02-24

**Authors:** Miwa Watanabe, Hiromichi Kosaka, Masamori Sugawara, Michihiro Maemoto, Yoko Ono, Takeshi Uemori, Ryota Shizu, Kouichi Yoshinari

**Affiliations:** ^1^ Research and Development Division Kyowa Kirin Co., Ltd. Shizuoka Japan; ^2^ Department of Molecular Toxicology, School of Pharmaceutical Sciences University of Shizuoka Shizuoka Japan

**Keywords:** DAX1, dihydroorotate dehydrogenase, Ewing's sarcoma, EWS‐FLI1, nucleotide pool

## Abstract

**Objective:**

EWS‐FLI1 is the most common oncogenic fusion protein in Ewing's sarcoma family tumors (ESFTs). DAX1, an orphan member of the nuclear receptor superfamily, is up‐regulated by EWS‐FLI1 and plays a key role in the transformed phenotype of ESFTs.

**Methods:**

To discover a functional inhibitor of DAX1 and EWS‐FLI1, we screened small‐molecular inhibitors using a DAX1 reporter assay system.

**Results:**

K‐234 and its derivatives, which were dihydroorotate dehydrogenase (DHODH) inhibitors, showed inhibitory effects in the reporter assay. K‐234 inhibited the growth of Ewing's sarcoma with various fusion types, and K‐234 derivatives altered the expression of EWS‐FLI1‐regulated genes. The DAX1 expression had no effect on the growth inhibitory effect of the K‐234 derivatives, while DHODH overexpression or uridine treatment attenuated their inhibitory effects, suggesting that inhibition by K‐234 derivatives occurs through DHODH inhibition. An in vivo study showed that a K‐234 derivative clearly inhibited tumor growth in an Ewing's sarcoma xenograft mouse model.

**Conclusion:**

Taken together, the present results suggest that DHODH inhibitors can inhibit the function of DAX1/EWS‐FLI1 in ESFTs and might be a therapeutic agent with potent anti‐tumor activity for Ewing's sarcoma patients.

## Graphical abstract

Dihydroorotate dehydrogenase exerts an anti‐tumor effect in mice bearing Ewing's sarcoma, and be identified as a therapeutic target for Ewing's sarcoma.

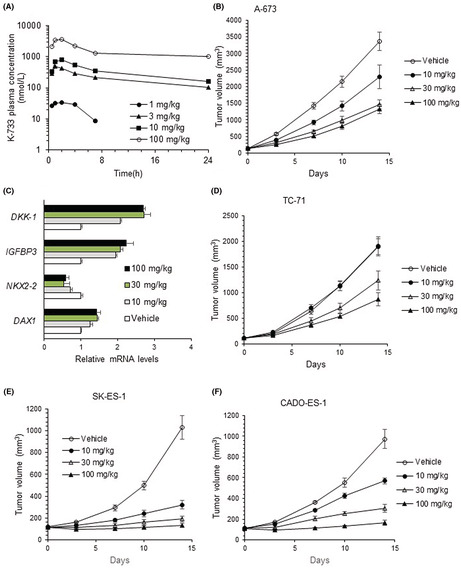

## 1 INTRODUCTION

Ewing's sarcoma is a highly malignant tumor that occurs in bones and/or soft tissues of children and young adults. Standard therapy for Ewing's sarcoma is surgery, radiation, and combined cytotoxic chemotherapy. There are no approved molecular‐targeted drugs.^1^ Although systemic chemotherapy has improved the survival of patients with local disease, the prognosis of patients with metastatic extraosseous or relapsed Ewing's sarcoma is very poor.^2^

All Ewing's sarcomas contain a fusion gene of the EWS and ETS family genes created by chromosomal translocation. Approximately 85% of Ewing's sarcomas contain the EWS‐FLI1 fusion gene due to the *t*(11;22)(q24;q12) translocation, and 10% have *t*(21;22)(q24;q12) translocation causing EWS‐ERG fusion.^1^, ^3^, ^4^ Both EWS‐FLI1 and EWS‐ERG fusion proteins work as oncogenic transcriptional factors, and the knockdown of EWS‐FLI1 in Ewing's sarcoma cell lines showed inhibitory effects on the cell proliferation, anchorage‐independent growth, and tumor growth in vivo in a xenograft mouse model.^5^, ^6^ EWS‐FLI1 is thus a promising therapeutic target for Ewing's sarcoma.

EWS‐FLI1 protein contains intrinsically disordered regions and has no catalytic domain;^7^ thus, EWS‐FLI1 has been considered a difficult therapeutic target. Recently, however, YK‐4‐279 and its derivative TK‐216 have been reported to inhibit the protein–protein interaction between EWS‐FLI1 and RNA helicase A, resulting in the cell growth inhibition of Ewing's sarcoma.^8^, ^9^

DAX1, also known as NR0B1, is an orphan member of the nuclear receptor superfamily.^10^ DAX1 has a unique structure: its C‐terminal region contains a ligand‐binding domain (LBD), as do other nuclear receptors, whereas the N‐terminal region has no DNA‐binding domain but contains 3.5 repeats of the LXXLL/LXXML motif, which plays important roles in binding to transcriptional coactivators or other proteins.^10^ Although the precise physiological role of DAX1 remains to be elucidated, DAX1 is reported to function as a corepressor or modulator for many transcriptional factors, such as SF1, ERR, PPAR, NR4A1, and Oct3/4.^11^, ^12^, ^13^, ^14^, ^15^

The promoter region of the DAX1 gene contains an EWS‐FLI1 binding sequence, and EWS‐FLI1 directly up‐regulates the DAX1 gene expression in Ewing's sarcomas.^16^, ^17^ In addition, it was reported that DAX1 was required for oncogenic functions, such as cell growth and oncogenic transformation, of EWS‐FLI1 in Ewing's sarcoma.^17^ The deletion of GGAA‐microsatellites in the *DAX1* promoter, which can serve as an EWS‐FLI1‐response element, exhibited impaired cell growth and the loss of colony formation in A‐673 cells.^6^ Furthermore, DAX1 protein directly interacted with EWS‐FLI1 in yeast two‐hybrid assays and coordinately regulated the expression of about 300 genes in Ewing's sarcomas.^17^ These results strongly suggest that DAX1 is one of the most important regulators of EWS‐FLI1 functions in terms of the progression of Ewing's sarcoma.

Based on these facts, we hypothesized that the chemical inhibition of DAX1 could modulate the growth and/or malignant progression of Ewing's sarcomas by inhibiting EWS‐FLI1 functions. To this end, we established an estrogen‐inducible DAX1 reporter assay system to obtain compounds showing DAX1 inhibition. Using this system, we screened small‐molecule compounds and identified dihydroorotate dehydrogenase (DHODH) inhibitors as inhibitors for DAX1/EWS‐FLI1 functions to reduce the growth of Ewing's sarcomas.

## 2 MATERIALS AND METHODS

### 2.1 Reagents

**FIGURE 1:**
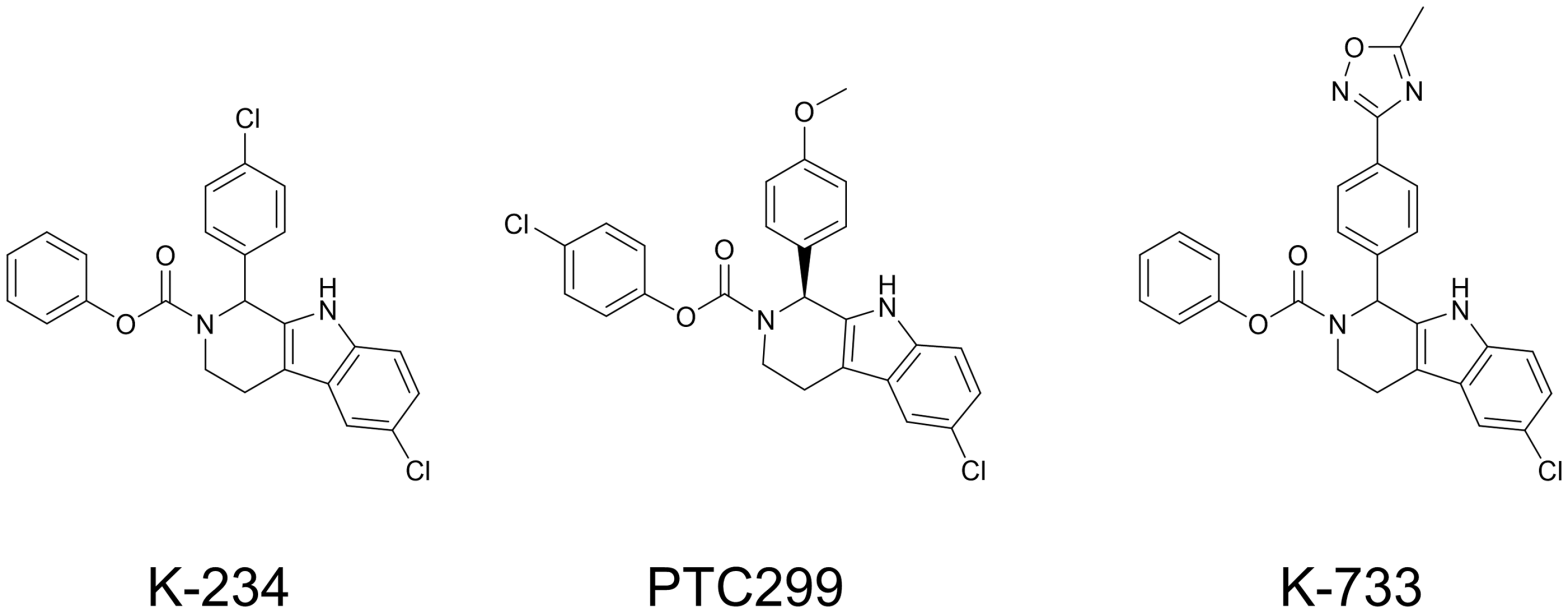
Chemical structures of K‐234, PTC299, and K‐733.

K‐234 (phenyl 6‐chloro‐1‐(4‐chlorophenyl)‐1, 3, 4, 9‐tetrahydro‐2*H*‐pyrido[3,4‐*b*]indole‐2‐carboxylate), PTC299 (4‐chlorophenyl (*S*)‐6‐chloro‐1‐(4‐methoxyphenyl)‐1,3,4,9‐tetrahydro‐2*H*‐pyrido[3,4‐*b*]indole‐2‐carboxylate), and K‐733 (phenyl 6‐chloro‐1‐(4‐(5‐methyl‐1,2,4‐oxadiazol‐3‐yl)phenyl)‐1,3,4,9‐tetrahydro‐2*H*‐pyrido[3,4‐*b*]indole‐2‐carboxylate) were synthesized by Kyowa Kirin (Shizuoka, Japan). Their chemical structures are shown in Figure 1. L‐Dihydroorotic acid was purchased from Sigma‐Aldrich (St. Louis, MO, USA). Orotic acid and 5‐fluoroorotic acid were purchased from Tokyo Chemical Industry (Tokyo, Japan). TransIT‐LT1 reagent was purchased from Mirus Bio (Madison, WI, USA). All other reagents were obtained from Tokyo Chemical Industry and FUJIFILM Wako Pure Chemical Industries (Osaka, Japan), or Sigma‐Aldrich, unless otherwise indicated.

### 2.2 Plasmid construction

The full‐length cDNA of human DAX1 (NM_000475.4) was subcloned into the pcDNA3.1 expression vector (Thermo Fisher Scientific, Waltham, MA, USA) (pcDNA3.1‐hDAX1) and transferred to a pAGal9 plasmid^18^ to obtain a human DAX1 expression plasmid (pAGal9p4‐hDAX1). DNA with 10 copies of the PPARγ‐response element (PPRE) derived from rat acyl‐CoA oxidase gene^19^ with a TATA sequence (Figure S1) and its antisense DNA was synthesized (Sigma‐Aldrich, St. Louis, MO, USA), annealed, and subcloned into a pACREplucGI plasmid (pAPPRE×10‐Luc+). The full‐length cDNA of human DHODH (NM_001361.4) was subcloned into the pcDNA3.1 hygro(+) (Thermo Fisher Scientific) (pcDNA3.1hygro(+)‐hDHODH).

### 2.3 Cell culture

A‐673, SK‐ES‐1, and AsPC‐1 cells were obtained from American Type Culture Collection (Manassas, VA, USA). TC‐71 and CADO‐ES1 cells were obtained from Leibniz Institute DSMZ ‐ German Collection of Microorganisms and Cell Cultures (Braunschweig, Germany). The details of cell culture media formulation and culture conditions are described in Materials and Methods S1.

### 2.4 Reporter gene assays

pAGal9‐hDAX1 and pAPPRE×10‐Luc + were transfected into KJMGER8 cells by electroporation using a Gene Pulser (Bio‐Rad Laboratories, Hercules, CA, USA), and clones stably expressing the DAX1 and luciferase reporter genes under the control of PPRE were selected. The obtained clone was termed KJMGER8/pAGal9p4‐hDAX1. KJMGER8/pAGal9p4‐hDAX1 cells (1.8 × 10^4^ cells/well) were seeded in 384‐well white plates (Greiner Japan, Tokyo, Japan) and cultured in RPMI1640‐IPTSG medium containing 10 nM 17β‐estradiol (E2; Sigma‐Aldrich), dissolved in ethanol, with test compounds at concentration ranges of 0.01 to 10,000 nM or vehicle (dimethyl sulfoxide [DMSO]). The final DMSO concentration was ≤0.3%, and the ethanol concentration was 0.1%. The next day, Blight‐Glo Luciferase Assay Reagent (Promega, Madison, WI, USA) was added to the medium. The luciferase activity was measured using a TopCount NXT system (PerkinElmer, Waltham, MA, USA). IC_50_ values were calculated using XLfit version 4 (ID Business Solutions, Surrey, UK).

### 2.5 Cell viability assays

AsPC‐1, A‐673, and TC‐71 cells were seeded in 96‐well plates (800 cells/well for AsPC‐1, 2000 cells/well for A‐673, and 400 cells/well for TC‐71) and cultured overnight. Vehicle (DMSO) or a test compound diluted with the medium was then added to the wells (final DMSO concentration was 0.3%), and 72 h later, 2,3‐bis[2‐methoxy‐4‐nitro‐5‐sulfophenynl]‐2*H*‐tetrazolium‐5‐carboxanilide inner salt (XTT) reagent (Roche Diagnostics, Mannheim, Germany) was added to the medium. After 3‐h incubation at 37°C, the formation of the formazan dye was determined by measuring absorbance at 490 and 650 nm using a SpectraMax 340PC system (Molecular Devices, Sunnyvale, CA, USA).

### 2.6 RNA interference and lipofection

RNA interference: small hairpin RNA (shRNA) targeting human DAX1 (5′‐TGCAGTGCGTGAAGTACATTC‐3′, TRCN0000413033) and small interfering RNA (siRNA) targeting human FLI1 (5′‐CGATCAGTAAGAATACAG‐3′) were purchased from Sigma‐Aldrich. shDAX1 was inserted into pLKO.1 (Sigma‐Aldrich). The plasmid was infected with A‐673 cells and cultured overnight, followed by selection with 0.25 μg/mL puromycin in DMEM. siFLI1 was transfected into A‐673 cells using Lipofectamine RNAiMAX transfection reagent (Thermo Fisher Scientific), and the cells were cultured in OPTI‐MEM (Sigma‐Aldrich). Twenty‐four hours later, the medium was removed and replaced with DMEM.

Lipofection: pcDNA3.1‐hDAX1 or pcDNA3.1hygro‐hDHODH was transfected to A‐673 cells using TransIT‐LT1 reagent according to the manufacturer's protocol. These A‐673 cells were subjected to hygromycin antibiotic selection for 6 days (hygromycin concentration, 700 μg/mL).

### 2.7 Quantitative reverse transcription polymerase chain reaction (RT‐qPCR)

Total RNA was extracted using the RNeasy Plus Mini kit (Qiagen, Valencia, CA, USA) according to the manufacturer's protocol. cDNA was synthesized from 1 μg of total RNA using a SuperScript VILO cDNA synthesis kit (Thermo Fisher Scientific). Real‐time PCR was carried out using TaqMan Fast Universal PCR Master Mix (Thermo Fisher Scientific). The primers used are listed in Table S1.

### 2.8 Animal experiments

All animal studies were performed in accordance with Standards for Proper Conduct sof Animal Experiments at Kyowa Kirin Co., Ltd., under the approval of the company's Institutional Animal Care and Use Committee. Male C.B17/Icr‐scid/scidJcl (SCID) mice (5 weeks old) were purchased from CREA Japan (Tokyo, Japan). K‐733 was suspended in 0.5% (w/v) methylcellulose 400 (MC400; FUJIFILM Wako Pure Chemical) and orally administered to mice once daily. Ewing's cells were suspended in Dulbecco's phosphate‐buffered saline (DPBS; Thermo Fisher Scientific, Waltham, MA, USA) and injected subcutaneously into the ventral side of mice at 5 × 10^6^ cells/0.1 mL/mouse for A‐673 and TC‐71 and 1 × 10^7^ cells/0.1 mL/mouse for CADO‐ES1 and SK‐ES‐1. In experiments using TC‐71 cells, 0.3 mg of anti‐asialo GM1 antibody (FUJIFILM Wako Pure Chemical) was injected intraperitoneally 1 day before TC‐71 injection. The tumor size and body weight were measured every 3 or 4 days. The tumor volume was calculated using the following equation:

$$\text{Tumor volume}\;\left({\mathrm{mm}}^{3}\right)=\mathrm{DL}\times \mathrm{DS}\times \mathrm{DS}\times 1/2,$$

where DL and DS represent the long diameter and short diameter, respectively. The pharmacokinetic analysis of K‐733 is described in Materials and Methods S2.

### 2.9 DHODH inhibition assays

The mitochondria fraction was isolated from A‐673 cells using Q proteome Mitochondria Isolation kit (QIAGEN, Hilden, Germany). The protein concentration of the fraction was determined using the DC‐protein assay kit (Bio‐Rad Laboratories, Hercules, CA, USA) with bovine serum albumin (Sigma‐Aldrich) as the standard. A portion of the mitochondria fraction (0.25 mg/mL, 40 μL) was pre‐incubated at 37°C for 2 min with a test compound in acetonitrile (final acetonitrile content, 0.625–1.25%) or acetonitrile only, and 10 μL of 5 nM L‐dihydroorotic acid was added, followed by further incubation at 37°C for 15 min (mitochondria fraction at 0.2 mg/mL in the assay condition). The reaction was quenched by adding 120 μL of 3 μM 5‐fluoroorotic acid acetonitrile solution. These samples were then centrifuged at 5000 *g* for 5 min to precipitate proteins, and the supernatant was collected. The samples were diluted 10‐fold with 70% acetonitrile‐water and subjected to liquid chromatography with tandem mass spectrometry (LCMSMS). The analyte was then separated on a hydrophilic interaction chromatography column (PC‐HILIC; 5 μm, 4.6 mm I.D. × 50 mm; Osaka Soda, Osaka, Japan). LCMSMS detection was performed by electrospray ionization in negative ion mode. The detected mass‐to‐charge ratios of orotic acid, L‐dihydroorotic acid, and 5‐fluoroorotic acid (internal standard) were 154.7/111.4 (Q1/Q3), 156.9/113.1, and 173.1/129.1, respectively.

### 2.10 Statistical analyses

The Kruskal‐Wallis test was performed using the SAS software program (Release 9.2; SAS Institute, Cary, NC, USA). A Pearson's correlation analysis was performed using the Microsoft Excel 2007 software program (Microsoft, Redmond, WA, USA). The level of significance was set at 5%.

## 3 RESULTS

### 3.1 Screening of DAX1 inhibitors

A DAX1 reporter assay system was constructed with the estrogen‐inducible Namalwa cell (KJMGER8 cell) by co‐transfection with pAGal9‐hDAX1 and pAPPRE×10‐Luc+.^18^ In the obtained cell line KJMGER8/pAGal9p4‐hDAX1, DAX1 expression is induced by E2 treatment, and the expressed DAX1 increases luciferase expression. Reporter activity was increased by E2 treatment as expected (Figure S2). When we expressed NR4A1 as an example of other nuclear receptors which is reported to interact with DAX1 in KJMGER8/pAGal9p4‐hDAX1 cells, E2 treatment did not affect the reporter activity (data not shown). These results suggest that the reporter activity depends on the DAX1 activation. Using this DAX1 reporter assay system, small‐molecule compounds in an in‐house library were screened to obtain DAX1 inhibitors, and K‐234 was identified (Figure 1). Around 300 K‐234 derivatives were then subjected to the reporter assay, and PTC299 and K‐733 were found to strongly inhibit reporter activity. The IC_50_ values for the inhibition by K‐234, PTC299, and K‐733 were determined to be 94.5, 33.0, and 9.01 nM, respectively. And it should be noted that the cytotoxic reagent paclitaxel did not show a remarkable inhibitory effect on the reporter activity (Figure S3).

### 3.2 Influences of DAX1 inhibitors on the growth of Ewing's sarcomas

**FIGURE 2:**
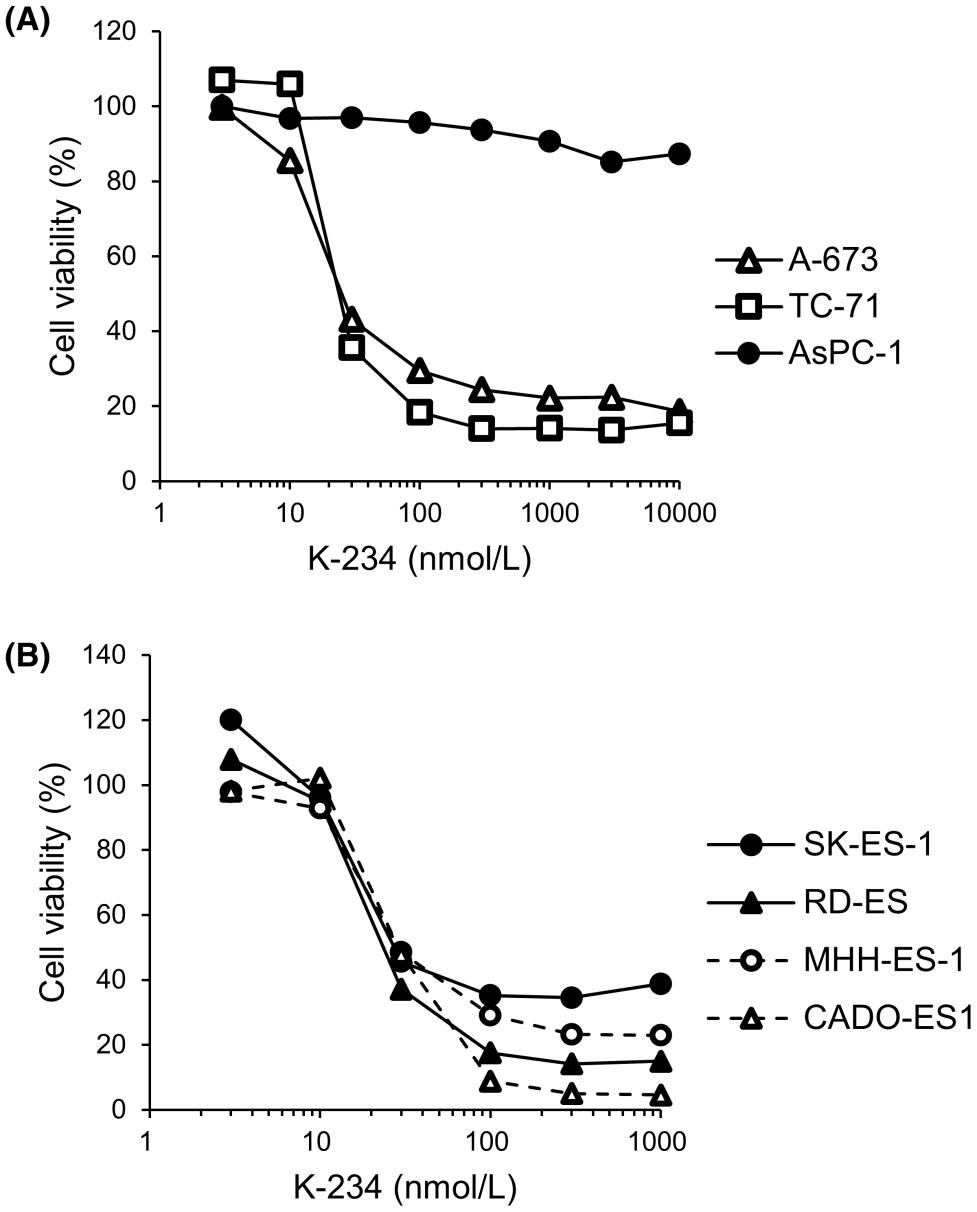
K‐234 influences the growth of Ewing's sarcoma. (A) Ewing's sarcoma‐derived A‐673 and TC‐71 cells and human pancreas adenocarcinoma‐derived AsPC‐1 cells as a control were treated with indicated concentrations of K‐234 for 72 h, and cell viability was measured. Each value represents the mean of duplicate measurements. (B) Ewing's sarcoma with type 2‐fusion gene‐derived SK‐ES‐1, RD‐ES, and MHH‐ES‐1 cells and Ewing's sarcoma with EWS‐ERG fusion gene‐derived CADO‐ES1 cells were treated with indicated concentrations of K‐234 for 120 h, and cell viability was measured. Each value represents the mean of duplicate measurements.

Next, we investigated the influence of K‐234 on Ewing's sarcoma cell growth. Ewing's sarcoma‐derived A‐673 and TC‐71 cells and human pancreas adenocarcinoma‐derived AsPC‐1 cells as a control, in which DAX1 protein was not detected in Western blotting (data not shown), were treated with K‐234 at various concentrations, and cell viability was determined (Figure 2A). The results demonstrated that the K‐234 treatment significantly reduced the viability of A‐673 and TC‐71 cells but not AsPC‐1 cells. PTC299, K‐733, K‐235 (a K‐234 derivative), and K‐522 (a different chemical structure from K‐234) also decreased the viability of these Ewing's sarcoma cells (Figure S4 and data not shown).

There are at least two types of the EWS‐FLI1 fusion gene: 1 and 2. Type 1 is a fusion of EWS exon 7 to FLI1 exon 6, which is the most common fusion found among EWS‐FLI1 fusion genes. Type 2 is a fusion of EWS exon 7 to FLI1 exon 5.^4^ The A‐673 and TC‐71 cells used above have the type 1 fusion.^20^ Therefore, we investigated the influence of K‐234 on the growth of other types of Ewing's sarcoma, including SK‐ES‐1, RD‐ES, and MHH‐ES‐1 cells with type 2 fusion and CADO‐ES1 cells the EWS‐ERG fusion.^21^ As shown in Figure 2A,B K‐234 treatment significantly reduced the viability of all cells tested. These results suggest that K‐234 inhibits the growth of Ewing's cells harboring various EWS‐ETS fusions.

### 3.3 Role of DAX1 in the growth inhibition of Ewing's sarcomas by K‐234 and its derivatives

**FIGURE 3:**
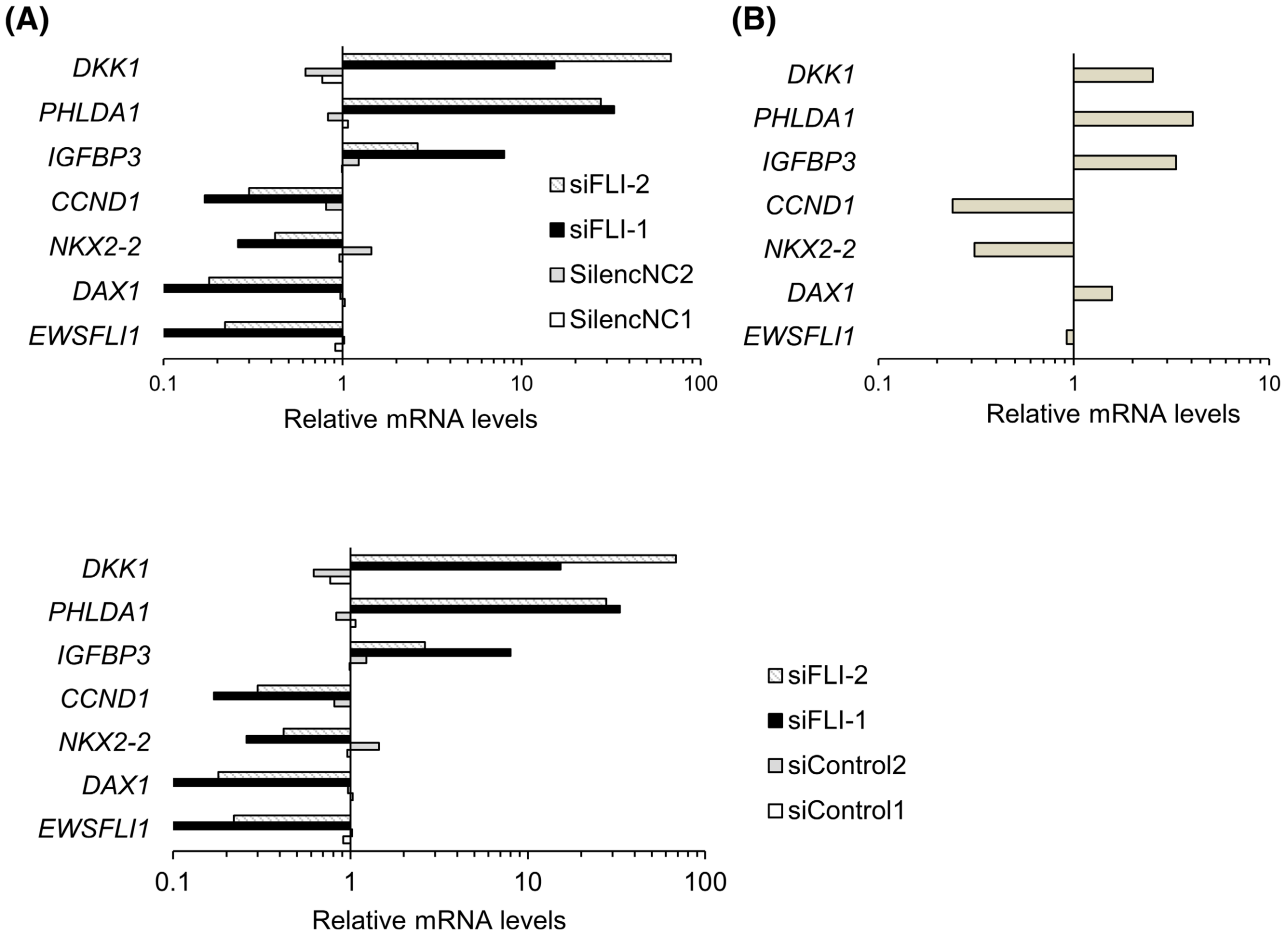
EWS‐FLI1‐related gene expression after treatment with DAX1 inhibitors and knockdown of FLI1 in A‐673 cells. (A) A‐673 cells were treated with siRNA of FLI1 (siFLI‐1, siFLI‐2) or control siRNA (control siRNA1, control siRNA2) for 24 h, and EWS‐FLI1‐related genes were analyzed (details of siRNA are shown in Table S2). The gene expression was normalized by the GAPDH expression and normalized by the mean of control siRNA‐treated samples. Each bar represents the mean of duplicate measurements. (B) A‐673 cells were treated with PTC299 at 1000 nM for 48 h, and EWS‐FLI1‐related genes were analyzed. Gene expression was normalized by the GAPDH expression and by DMSO‐treated samples. Each bar represents the mean of duplicate measurements.

To investigate the role of DAX1 in the reduction in Ewing's sarcoma cell viability by K‐234 and its derivatives, we compared the EWS‐FLI1‐related gene expression after EWS‐FLI1 knockdown with FLI1‐targeting siRNA (Figure 3A) and after treatment with PTC299 (Figure 3B). Since DAX1 expression is positively regulated by EWS‐FLI1 in Ewing's sarcoma, we decided to knock down FLI1 instead of DAX1 to assess the gene signature profiles clearly.

Except for the DAX1 gene, treatment with PTC299 and that with FLI1 siRNA showed similar effects on EWS‐FLI1 target gene expression (Figure 3A,B). PTC299 treatment slightly induced DAX1 expression. It is reported that a DAX1‐binding site is present in the *DAX1* promoter, and *DAX1* expression can be negatively regulated by DAX1 itself.^22^ Therefore, DAX1 induction by PTC299 might be related to the functional inhibition of DAX1. These results suggest that PTC299 inhibits Ewing's sarcoma cell growth by functional inhibition of DAX1 and EWS‐FLI1.

**FIGURE 4:**
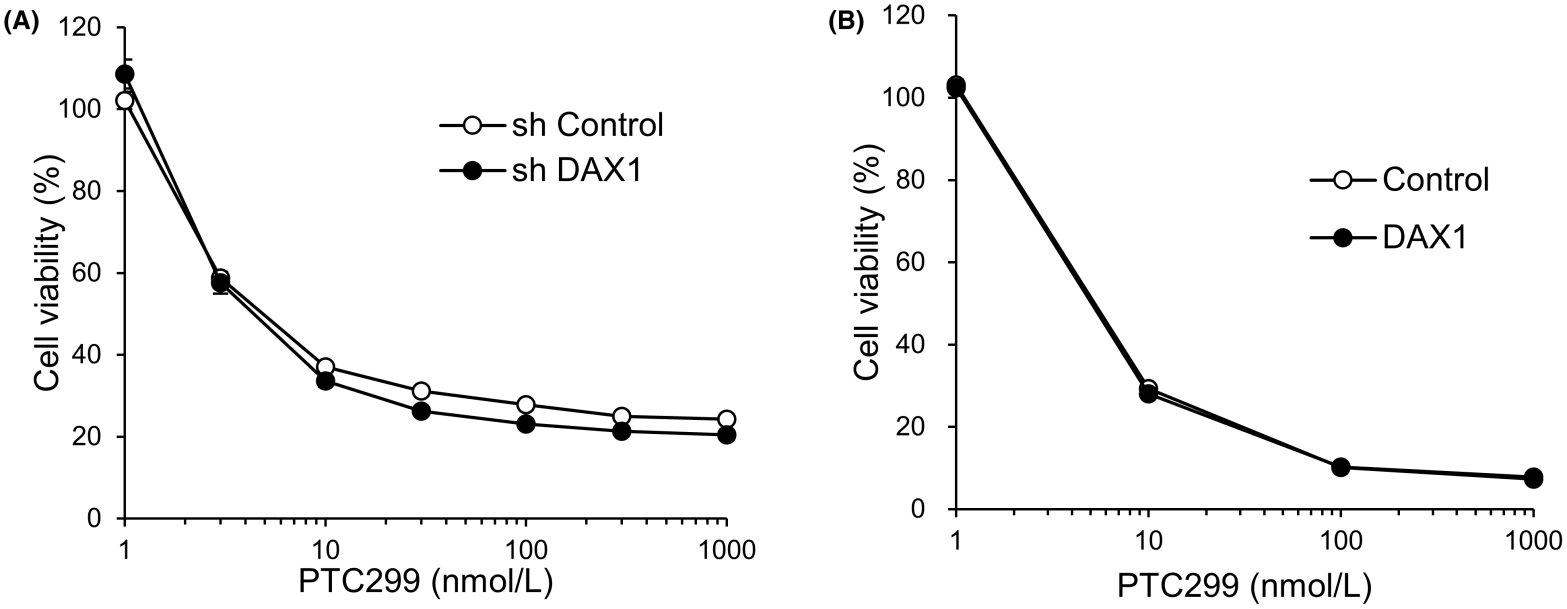
Influence of knockdown or overexpression of DAX1 on Ewing's sarcoma cell growth. (A) A‐673 cells were infected with DAX1 or control shRNA‐expressing lentivirus. The A‐673 cells knocked down with DAX1 and control A‐673 cells were treated with indicated concentrations of PTC299 for 72 h, and cell viability was measured. Each plot represents the mean of triplicate measurements. (B) A‐673 cells were infected with DAX‐1 or control‐expressing lentivirus. The A‐673 cells with DAX1 or control A‐673 cells were treated with indicated concentrations of PTC299 for 72 h, and cell viability was measured. Each plot represents the mean of triplicate measurements.

We further evaluated the effects of the knockdown and overexpression of DAX1 on the PTC299‐dependent reduction in the viability of A‐673 cells, noting no marked effect on the reduction of the viability by PTC299 treatment (Figure 4A,B). These results suggest that DAX1 is not a direct target of K‐234 or its derivatives and that the compounds indirectly inhibit DAX1 in Ewing's sarcomas.

### 3.4 Identification of DHODH as a possible target of K‐234 and its derivatives

**FIGURE 5:**
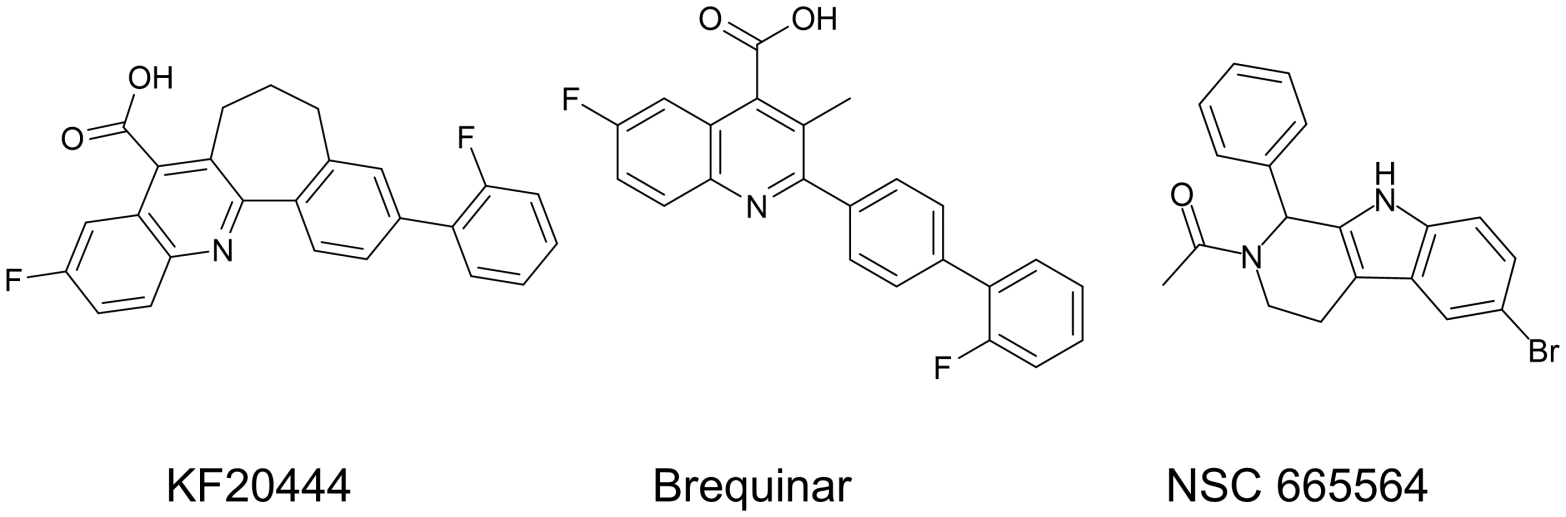
Chemical structure of KF20444, Brequinar, and NSC 665564.

To identify a target protein of K‐234, we ran a structure search using SciFinder to identify compounds with a structure similar to K‐234 and identified the DHODH inhibitor NSC 665564 (Figure 5).^23^ DHODH catalyzes the oxidation of dihydroorotate to orotate in the de novo pyrimidine biosynthesis pathway, resulting in the depletion of pyrimidine derivatives, such as UTP and UMP.^23^

**FIGURE 6:**
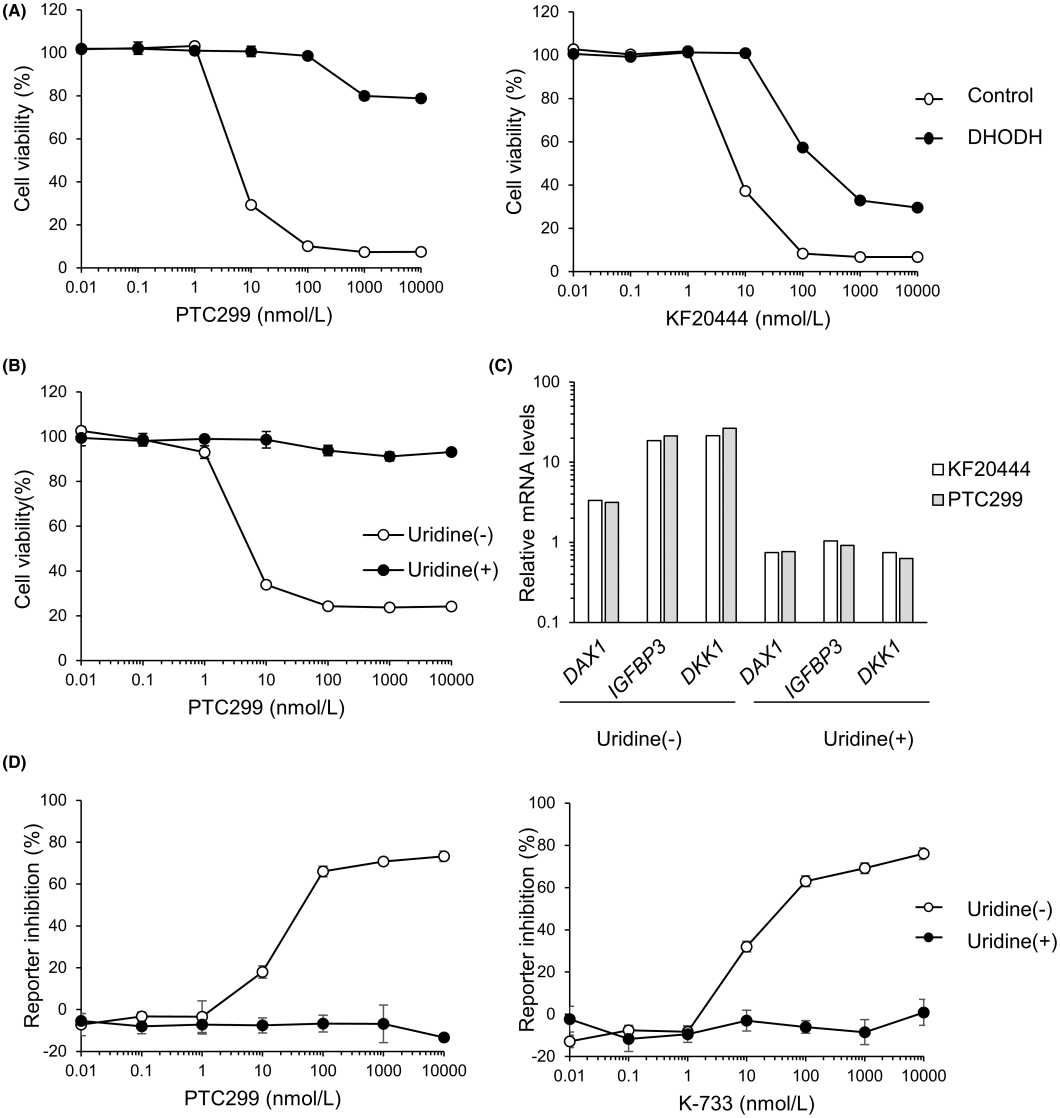
Influence of DHODH overexpression on Ewing's sarcoma cell growth and the EWS‐FLI1‐regulated gene expression. (A) pcDNA3.1hygro(+)‐hDHODH was transfected into A‐673 cells. The A‐673 cells with DHODH or control A‐673 cells were treated with indicated concentrations of PTC299 and KF20444 for 72 h, and cell viability was measured. Each plot represents the mean of triplicate measurements. (B) A‐673 cells with or without uridine (100 μM) were treated with indicated concentrations of PTC299 for 72 h, and cell viability was measured. Each plot represents the mean of triplicate measurements. (C) A‐673 cells with or without uridine (100 μM) were treated with PTC299 and KF20444 at 1000 nmol/L for 48 h, and the EWS‐FLI1‐related gene expression was evaluated. The expression was normalized by GAPDH and by DMSO‐treated samples. (D) KJMGER8/pAGal9p4‐hDAX1 cells with or without uridine (100 μM) were treated with indicated concentrations of PTC299 and K‐733 overnight, and the luciferase activity was measured. Each plot represents the mean of quadruplicate measurements.

To investigate whether or not K‐234 and its derivatives could reduce the cell viability of Ewing's sarcoma by interacting with DHODH, we evaluated the effects of DHODH overexpression on the viability of A‐673 cells. The cell growth inhibitory effect of PTC299 was significantly attenuated by DHODH overexpression in the cells (Figure 6A). We then investigated whether or not KF20444,^24^ another DHODH inhibitor, reduced the viability of A‐673 cells with or without DHODH overexpression. Surprisingly, KF20444 reduced the cell viability of A‐673 cells to a similar extent as PTC299, and the reduction was weakened by DHODH overexpression (Figure 6A, right panel).

To confirm the role of DHODH in the PTC299‐mediated inhibition of Ewing's sarcoma cell growth, we investigated the effect of uridine addition on the viability of PTC299‐treated A‐673 cells. Uridine is a metabolite of pyrimidine biosynthesis, and its addition abrogated the pyrimidine starvation induced by DHODH inhibitors.^25^ These results demonstrated that the growth inhibitory effect of PTC299 was completely blocked by uridine addition (Figure 6B).

Next, we investigated the influences of KF20444 on the expression of EWS‐FLI1 target genes, namely *DAX1*, *IGFBP3*, and *DKK1*, in A‐673 cells and compared the profile to that induced by PTC299. The results demonstrated that KF20444 treatment up‐regulated the expression of these genes, as did PTC299 (Figure 6C). We thus evaluated the influences of uridine addition on the gene expression changes induced by KF20444 and PTC299. Both KF20444‐ and PTC299‐mediated up‐regulation of DAX1, IGFBP3, and DKK1 was abrogated by uridine addition (Figure 6C). Similar results were obtained with K‐733 (data not shown).

We then investigated the influences of uridine addition on the inhibitory effects of PTC299 and K‐733 on DAX1 reporter activity. As expected, the PTC299 and K‐733‐mediated inhibition was completely abrogated by uridine addition (Figure 6D, and data not shown).

**FIGURE 7:**
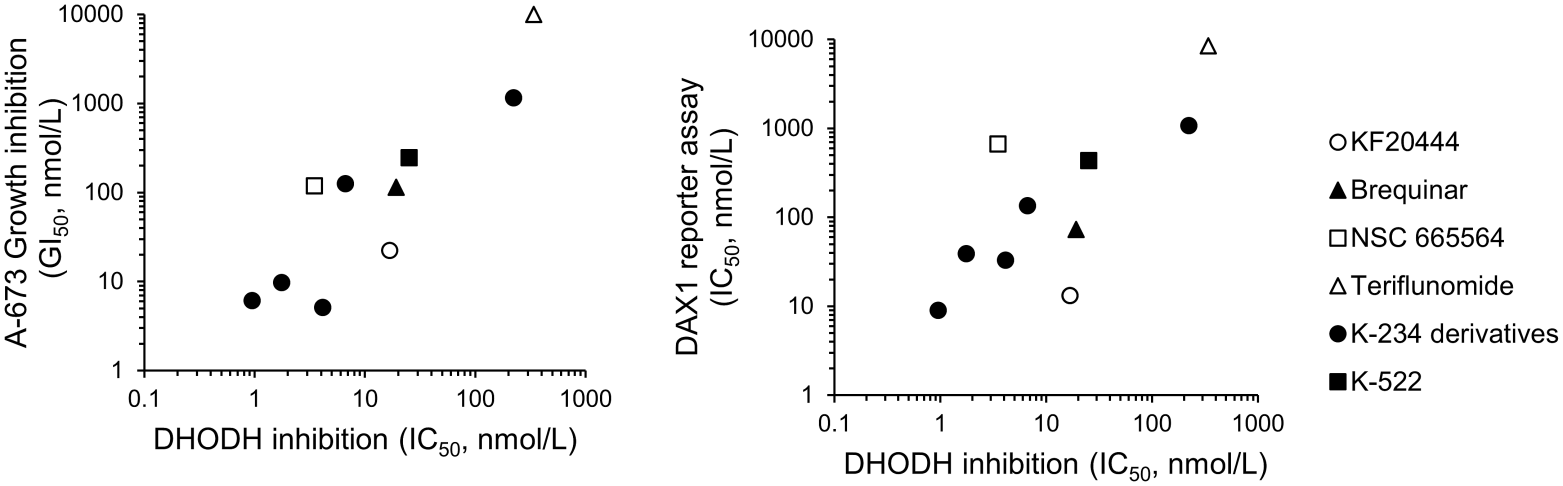
DHODH IC_50_ correlates A‐673 GI_50_ and DAX1 reporter IC_50_. IC_50_ values against DHODH activity were obtained for K‐234 derivatives and DHODH inhibitors. The DHODH IC_50_ values were compared with their GI_50_ values for A‐673 cells and with the DAX1 reporter IC_50_ values. The A‐673reporter GIC_50_ values of Teriflunomide were shown as 10,000 nM although it was above 10,000 nM.

To confirm the role of DHODH inhibition in the reduced viability of Ewing's sarcomas induced by K‐234 and its derivatives, we investigated the inhibitory effects of these compounds against DHODH enzymatic activity and compared its 50% inhibitory concentration (IC_50_) values with their 50% growth inhibition concentration (GI_50_) values for A‐673 cells. PTC299 and K‐733 strongly inhibited orotic acid production through DHODH, with IC_50_ values of 4.13 and 0.953 nM, respectively. Known DHODH inhibitors, including NSC 665564, teriflunomide, brequinar, and KF20444, were also assessed for their inhibitory effects in this assay, and the IC_50_ values were comparable to those reported previously.^26^, ^27^, ^28^ On comparing the IC_50_ and GI_50_ values obtained for K‐234 derivatives, including PTC299 and K‐733, and the DHODH inhibitors, a good correlation was found (Figure 7A). We also determined the IC_50_ values for a DAX1 reporter assay of these compounds and found that the values were also well‐correlated with the IC_50_ values for orotic acid production by DHODH (Figure 7B; Table S3).

Taken together, these results strongly suggest that K‐234 and its derivatives identified as DAX1 inhibitors inhibit DHODH enzymatic activity to reduce the growth of Ewing's sarcomas.

### 3.5 in vivo antitumor effects of K‐733

**FIGURE 8:**
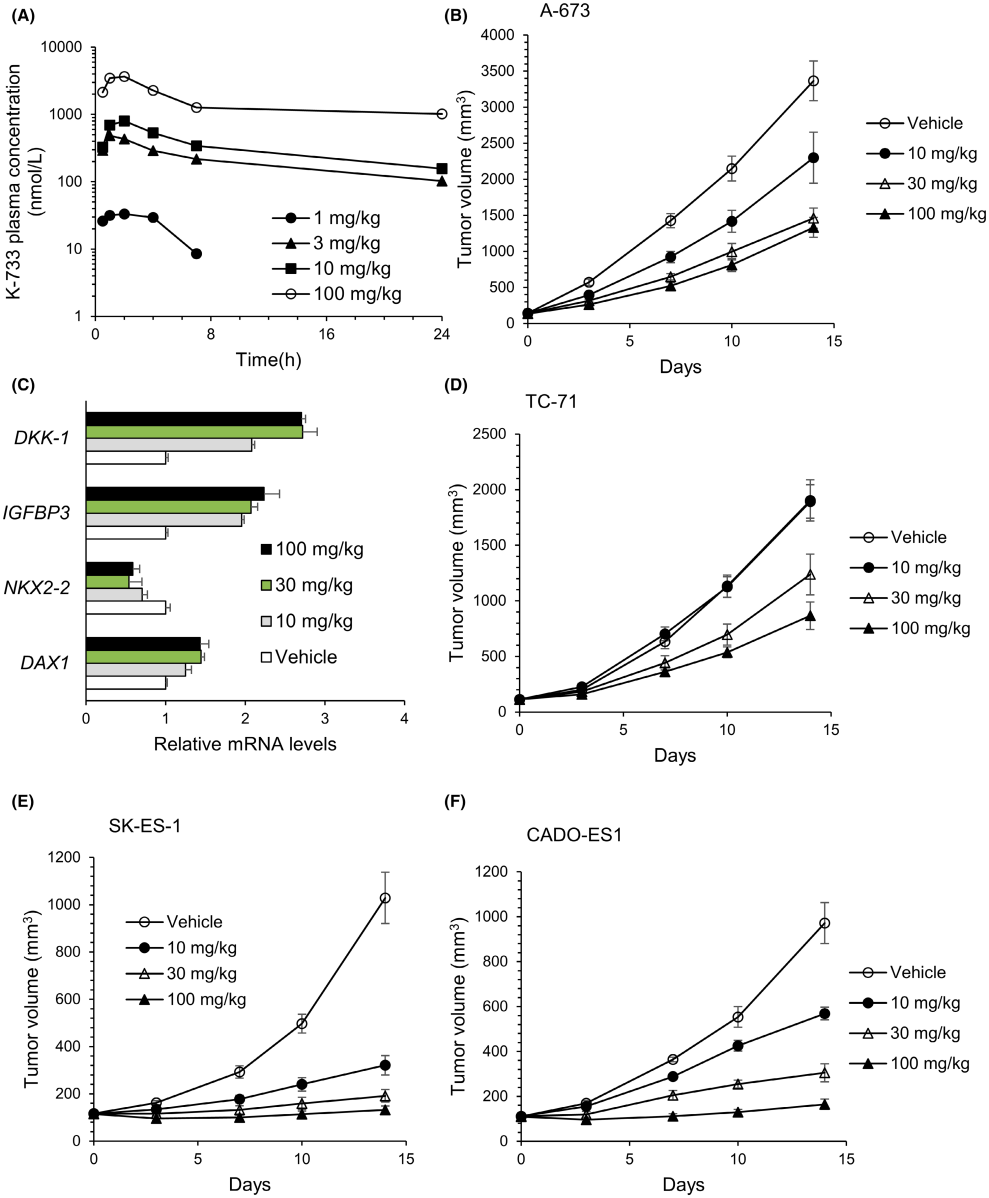
K‐733 exerts an anti‐tumor effect in mice bearing Ewing's sarcoma. (A) Plasma concentration‐time profiles of K‐733 in mice after a single oral administration of K‐733. Each plot represents the mean of duplicate measurements. (B) Antitumor effect of K‐733 on A‐673 cells. Indicated doses of K‐733 were orally administered once daily for 14 days. Each plot represents the mean of *n* = 5 and SE. C, Influence of K‐733 on EWS‐FLI1‐related genes. Tumors were sampled after 14 days oral administration of K‐733. Each plot represents the mean of *n* = 5 and SE. (D, E, F) Antitumor effects of K‐733 on TC‐71, SK‐ES133, and CADO‐ES‐1 cells. Indicated doses of K‐733 were orally administered once daily for 14 days. Each plot represents the mean of *n* = 5 and SE.

Finally, we investigated whether or not K‐733, which showed the lowest IC_50_ for the DAX1 reporter assay, exerted antitumor activity in vivo. First, we evaluated the plasma concentration‐time profile of K‐733 after single oral administration at dose ranges from 1 to 100 mg/kg in BALB/c mice (donors of SCID mice) for dose selection (Figure 8A). The plasma concentration of K‐733 increased dose‐dependently, and at ≥10 mg/kg, the K‐733 plasma concentration was higher than the IC_50_ value obtained in the DAX1 reporter assay and for DHODH inhibition. Therefore, we selected dose ranges from 10 to 100 mg/kg for in vivo studies.

Antitumor effects of K‐733 were evaluated in a mouse xenograft model bearing Ewing's sarcomas. When K‐733 was orally administered to mice bearing A‐673 cell‐derived tumors at 10, 30, or 100 mg/kg once daily for up to 14 days, the tumor volume was reduced without a body weight loss (Figure 8B and data not shown). The greatest change in treatment per control ratio (T/C: 0.38) was observed at 100 mg/kg. After 14‐day treatment, tumors were harvested, and EWS‐FLI1 target gene expression was analyzed (Figure 8C). K‐733 treatment decreased the mRNA levels of *NKX2‐2* and increased those of *IGFBP3* and *DKK1*. This pattern was similar to that observed with FLI1‐knockdown in A‐673 cells (Figure 3). K‐733 treatment had no marked effect on the *DAX1* mRNA levels in the tumors.

Next, we assessed whether or not K‐733 inhibited the tumor growth of other types of Ewing's sarcomas, including TC‐71 with the type 1 fusion gene, SK‐ES‐1 with the type 2 fusion gene, and CADO‐ES1 with the EWS‐ERG fusion gene. K‐733 was orally administered to mice at 10, 30, or 100 mg/kg once daily, and tumor sizes were evaluated. The growth of all the tumors tested was reduced dose‐dependently, although TC‐71‐derived tumors were less sensitive to K‐733 than others (Figure 8).

## 4 DISCUSSION

In this study, we constructed a DAX1 reporter assay system, in which DAX1 expression is induced by estrogen treatment and DAX1 induces reporter gene expression through PPARγ‐responsive element (PPRE)s (Figure S2). Using this reporter assay system, we screened small‐molecule compounds and found that K‐234 and its derivatives strongly inhibited the DAX1‐dependent reporter gene expression.

In in vitro assays, K‐234 inhibited the growth of Ewing's sarcomas with various fusion types, including A‐673 and TC‐71 with the type 1 fusion of EWS‐FLI1, SK‐ES‐1, and RE‐ES with the type 2 fusion, and MHH‐ES‐1 and CADO‐ES1 with EWS‐ERG (Figure 2A,B). The compounds did not inhibit the growth of pancreas adenocarcinoma‐derived AsPC‐1 cells. Consistently, K‐733 showed dose‐dependent inhibition of tumor growth in mice bearing various types of Ewing's sarcoma, including A‐673, TC‐71, SK‐ES‐1, and CADO‐ES‐1. (Figure 8B,D). We also found that treatment of A‐673 cells with PTC299 altered the expression of EWS‐FLI1‐related genes, including *CCND1*, *NKX2.2*, *DAX1*, *DKK1*, *PHLDA1*, and *IGFBP3*,^29^, ^30^, ^31^, ^32^ with a similar profile to that obtained after treatment with siRNA targeting *FLI1* (Figure 3A,B). In A‐673‐bearing mice, K‐733 treatment dose‐dependently changed the expression of EWS‐FLI1‐related genes in tumors (Figure 8C). Taken together, these results suggest that K‐234 derivatives inhibit the growth of Ewing's sarcoma in vitro and in vivo through EWS‐FLI1.

The present study showed that PTC299 reduced the viability of A‐673 cells, regardless of the DAX1 expression (i.e. overexpression or shRNA‐mediated knockdown) (Figure 4). *DAX1* mRNA levels were reduced to 11% compared to controls after shDAX1 treatment in A‐673 cells (Figures S3 and S5). EWS‐FLI1 directly binds to the GGAA‐rich region of the *DAX1* promoter and up‐regulates the *DAX1* expression, which is associated with Ewing's sarcoma cell growth.^6^ Therefore, knockdown of *DAX1* was expected to reduce cell proliferation, while DAX1 overexpression was expected to increase the proliferation of A‐673 cells. However, PTC299 reduced the viability of A‐673 cells regardless of the DAX1 expression, suggesting the negligible contribution of DAX1 to the inhibitory effect of A‐673 cell proliferation by PTC299.

By a structural similarity search with K‐234, we found that NSC 665564, which is reportedly a DHODH inhibitor, has a similar structure to K‐234 derivatives. We also found that K‐733 and PTC299 strongly inhibited orotate formation with very low IC_50_ values. Prior to our present report, Cao et al. demonstrated that PTC299 inhibited VEGF production and cell proliferation, which was linked to the inhibition of DHODH^33^. Furthermore, the IC_50_ values for the DAX1 reporter assay were significantly correlated with those for the DHODH enzymatic assay for DHODH inhibitors and K‐234 derivatives (Figure 7). These results strongly suggest that DHODH is the actual target of the K‐234 derivatives.

To further investigate whether or not DHODH was a target of the K‐234 derivatives, we evaluated the influence of DHODH overexpression on the inhibitory effect of PTC299 on A‐673 cell growth. Since DHODH inhibitors have been reported to exhibit cytotoxicity by depleting UTP, we investigated the influence of uridine supplementation on the inhibitory effect of PTC299 on A‐673 cell growth. We demonstrated that the growth inhibition was canceled by DHODH overexpression as well as uridine addition. Furthermore, the PTC299‐induced changes in EWS‐FLI1‐related gene expression were also canceled by adding uridine (Figure 6A–C). These results suggest that the inhibitory effect on Ewing's sarcoma cell proliferation by K‐234 derivatives involves DHODH inhibition.

DHODH catalyzes the oxidation of dihydroorotate to orotate, which is further phosphorylated to UMP and finally to UTP. Pyrimidine synthesis involves a de novo pathway via DHODH and a salvage pathway that recycles nucleotides from extracellular uridine and cytidine. Rapidly proliferating cells, such as leukemias and lymphomas, require more pyrimidine and are more sensitive to DHODH inhibition than normal cells, and are dependent on the de novo pathway. Indeed, the DHODH inhibitor leflunomide has been reported to arrest T cells in the G1 phase by UTP depletion in activated lymphocytes, resulting in the inhibition of T cell proliferation.^34^ PTC299 has been developed for AML after the identification of DHODH as its target, although it was originally developed for solid tumors.^35^ Taken together, Ewing's sarcoma is expected to show high dependence on de novo pyrimidine synthesis and be sensitive to DHODH inhibition.

Not only the K‐234 derivatives but also KF20444 altered the expression of EWS‐FLI1‐related genes in A‐673 cells (Figure 6C). PTC299 reportedly inhibits the translation of *VEGF* mRNA, and the inhibitory effect is considered to be a downstream effect of DHODH inhibition.^33^ Although the detailed relationship between VEGF translation inhibition and DHODH inhibition is unknown, uridine depletion from the nucleic acid pool may affect stress‐responsive transcription factors in non‐normal tissues. As EWS‐FLI1 is also a transcription factor, EWS‐FLI1 expression may be affected by the change in the nucleic acid pool. Further study is needed to understand the relationship between changes in the expression of EWS‐FLI1‐related genes and de novo pyrimidine synthesis. As well as Ewing's sarcoma, synovial sarcoma is classified as a soft‐tissue sarcoma and is reported to be caused by a chromosomal abnormality, and thus it might be worth evaluating the effect of DHODH inhibitor on synovial sarcoma.

K‐733 inhibited tumor growth at 30 mg/kg in A‐673‐bearing mice without affecting body weight gain up to 100 mg/kg. However, this compound showed a 20‐fold higher IC_50_ value for the mouse liver mitochondrial fraction than that for A‐673 mitochondrial fraction (Table S4). There are species differences in the inhibitory activity of the DHODH inhibitors leflunomide and brequinar.^27^, ^28^ For example, in contrast with K‐733, teriflunomide, an active form of leflunomide, showed a 50‐fold higher IC_50_ value for DHODH in humans than for mice and rats than for humans in vitro. These facts suggest that K‐733 can inhibit human‐derived tumor growth at doses that do not affect normal mouse cells, resulting in a wide safety window.

The changes in EWS‐FLI1‐related gene expression by KF20444 treatment were canceled by uridine addition; however, the inhibition of A‐673 cell proliferation by KF20444 was partially attenuated by DHODH overexpression (Figure 6A,C), whereas those changes induced by PTC299 were completely canceled by uridine addition or DHODH overexpression (Figure 6A,B). These results suggest that KF20444 inhibits A‐673 cell proliferation by both DHODH inhibition and other unknown mechanisms (while PTC299 does so by DHODH inhibition alone) and that PTC299 is a more selective DHODH inhibitor than KF20444.

In conclusion, we demonstrated that K‐234 and its derivatives, which were identified using the DAX1 reporter assay, can inhibit DHODH, resulting in changes in the EWS‐FLI1‐related gene expression and the inhibition of Ewing's sarcoma cell growth in vitro and in vivo. To our knowledge, this is the first report of DHODH inhibitors, including K‐234 and its derivatives, being viable drug candidates for the treatment of various types of Ewing's sarcoma.

## AUTHOR CONTRIBUTIONS

**Miwa Watanabe:** Conceptualization (equal); data curation (equal); investigation (equal); methodology (equal); validation (equal); visualization (equal); writing – original draft (lead). **Hiromichi Kosaka:** Conceptualization (equal); data curation (equal); methodology (equal); validation (equal); writing – review and editing (supporting). **Masamori Sugawara:** Methodology (equal). **michihiro maemoto:** Methodology (equal). **Yoko Ono:** Methodology (equal). **Takeshi Uemori:** Data curation (equal). **Ryota Shizu:** Writing – review and editing (supporting). **Kouichi Yoshinari:** Writing – review and editing (lead).

## CONFLICT OF INTEREST STATEMENT

Miwa Watanabe, Hiromichi Kosaka, Masamori Sugawara, Michihiro Maemoto, Yoko Ono, and Takeshi Uemori are employees of Kyowa Kirin Co., Ltd.

## ETHICS STATEMENT

Approval of the research protocol by an Institutional Reviewer Board: N/A. Informed Consent: N/A. Registry and the Registration No. of the study/trial: N/A. Animal Studies: All animal studies were performed in accordance with Standards for Proper Conduct of Animal Experiments at Kyowa Kirin Co., Ltd. under the approval of the company's Institutional Animal Care and Use Committee.

## Supporting information


**Appendix S1.** Supporting Information

## Data Availability

The data that support the findings of this study are available from the corresponding author upon reasonable request.
