# Supplementary material for: Screening for DAX1/EWS‐FLI1 functional inhibitors identified dihydroorotate dehydrogenase as a therapeutic target for Ewing's sarcoma
Source: Cancer Med. 2023 Feb 24;12(8):9802–14. doi: 10.1002/cam4.5741 (PMC10166890; doi:10.1002/cam4.5741)
Supplement: Supplementary file 1 — Appendix S1. Supporting Information [file CAM4-12-9802-s001.docx]

Table S1. Primer list.

| Gene | Primer |
| --- | --- |
| EWSFLI1 | Hs03024497_f1 |
| CCND1 | Hs00765553_m1 |
| PHLDA1 | Hs00705810_s1 |
| IGFBP3 | Hs00181211_m1 |
| UBC | Hs00824723_m1 |
| HPRT1 | Hs02800695_m1 |
| NR0B1 | Hs00230864_m1 |
| DKK1 | Hs00183740_m1 |
| NKX2-2 | Hs00159616_m1 |
| GAPDH | Fw: 5’-ACAGTCAGCCGCATCTTCTTT-3’  Rv: 5’-CCCAATACGACCAAATCCGT-3’  Probe: 5’-CGAGCCACATCGCTCAGACACCAT-3’ |

Table S2. siRNA sequences

| Gene | siRNA | |
| --- | --- | --- |
| FLI1-1 | Target sequence | 5’-CGATCAGTAAGAATACAGA-3’ |
|  | 3’-overhang | dTdT/dTdT |
|  | Sense | 5’-CGAUCAGUAAGAAUACAGAdTdT-3’ |
|  | Anti-sense: | 5’-UCUGUAUUCUUACUGAUCGdTdT-3’ |
| FLI1-2 | Target sequence | 5’-CAGCCACATCCGACCGAGT-3’ |
|  | 3’-overhang | dTdT/dCdA |
|  | Sense | 5’-CAGCCACAUCCGACCGAGUdTdT-3’ |
|  | Anti-sense | 5’-ACUCGGUCGGAUGUGGCUGdCdA-3’ |
| Control #1 | Silencer Select Negative Control #1 (Thermo Fisher scientific) | |
| Control #2 | Silencer Select Negative Control #2 (Thermo Fisher scientific) | |

Table S3. IC_50_ values for DHODH, A673 GI_50_, and IC_50_ for DAX1 reporter assay

| Compound | DHODH IC_50_  (nmol/L) | A673 GI_50_  (nmol/L) | DAX1 reporter IC_50_  (nmol/L) |
| --- | --- | --- | --- |
| KF20444 | 16.8 | 22.2 | 13.2 |
| Teriflunomide | 339 | >10000 | 8498 |
| Brequinar | 19.1 | 115 | 73.0 |
| NSC 665564 | 3.49 | 119 | 666 |
| PTC299 | 4.13 | 5.11 | 33.0 |
| K-785 | 223 | 1157 | 1080 |
| K-733 | 0.953 | 6.11 | 9.01 |
| K-203 | 1.76 | 9.8 | 39.0 |
| K-356 | 6.65 | 126 | 136 |
| K-522 | 25.2 | 247 | 437 |

Table S4. IC_50_ values for DHODH inhibition with mouse, rat, and monkey liver mitochondria.

| Compound | DHODH IC_50_ | | |
| --- | --- | --- | --- |
|  | Mouse  (nmol/L) | Rat  (nmol/L) | Monkey  (nmol/L) |
| KF20444 | 6.10 | 34.1 | 4.41 |
| Brequinar | 4.90 (57^a^) | 56.8 (127^a^, 367^b^) | 7.22 |
| Teriflunomide | 5.84 (82^a^) | 6.57 (18^a^) | 75.5 |
| PTC299 | 103 | 1520 | 0.722 |
| K-733 | 120 | NT | NT |
| K-203 | 77.9 | 712 | 16.9 |

NT, not tested.

Values in parentheses are from the literature.

a, Ullrich A, Knecht W, Fries M, Loffler M. Recombinant expression of N-terminal truncated mutants of the membrane bound mouse, rat and human flavoenzyme dihydroorotate dehydrogenase. *Eur J Biochem*. 2001, 68: 1861-1868.

b, Knecht W, Henseling J, Loffler M. Kinetics of inhibition of human and rat dihydroorotate dehydrogenase by atovaquone, lawsone derivatives, brequinar sodium and polyporic acid. *Chem Biol Interact*. 2000, 124: 61-76.

Supplementary Figures.

CCTGCAggaggacaaaggtcatt aggacaaaggtcagg aggacaaaggtcacc aggacaaaggtcaa aggacaaaggtcacg ttctcggg aggacaaaggtcatt aggacaaaggtcagg aggacaaaggtcacc aggacaaaggtcaa aggacaaaggtca gttctcgcgaggg TATATAAT ggatctcgagcccgggggtaccctaggctttgaaaaagcttggcattccggtactgttggtaaagccacc ATGGAAGACG

PPRE x 5

PPRE x 5

TATA

Luc

Figure S1. The structure of the reporter plasmid.

DNA with 10 copies of the PPARγ-response element (PPRE) derived from the rat acyl-CoA oxidase gene, 5’- aggacaaaggtca-3’ with a TATA sequence was synthesized (Sigma-Aldrich), annealed, and subcloned into a pACREplucGI plasmid to prepare pAPPRE×10-Luc+.

Figure S2. Reporter activity is activated by E2 treatment in the DAX1 reporter assay system.

Namalwa cells (KJMGER8 cell) co-transfected with pAGal9-hDAX1 and pAPPRE×10-Luc+ were cultured with or without 10 nM 17β-estradiol (E2) for 24 h, and then the luciferase activity was measured. Bars represent means ± SD (n=3).

Figure S3. Paclitaxel did not inhibit DAX1 reporter activity.

KJMGER8/pAGal9p4-hDAX1 cells were treated with indicated concentrations of paclitaxel overnight, and the luciferase activity was measured. Each plot represents the mean of duplicate measurements.

S4A

S4B

S4C


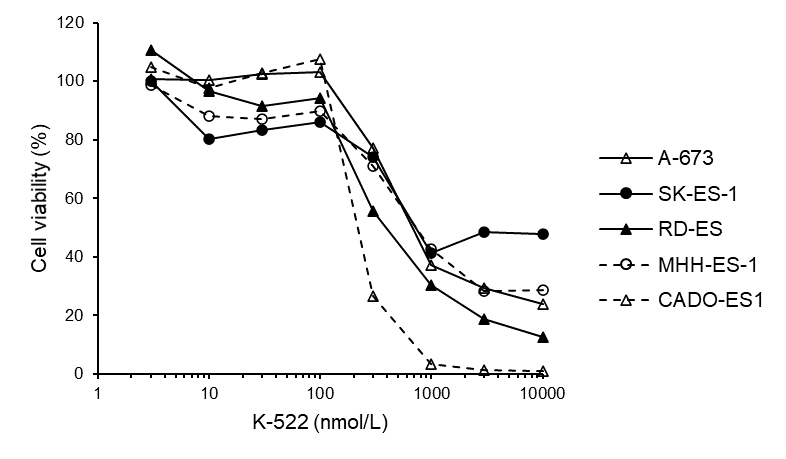


S4D


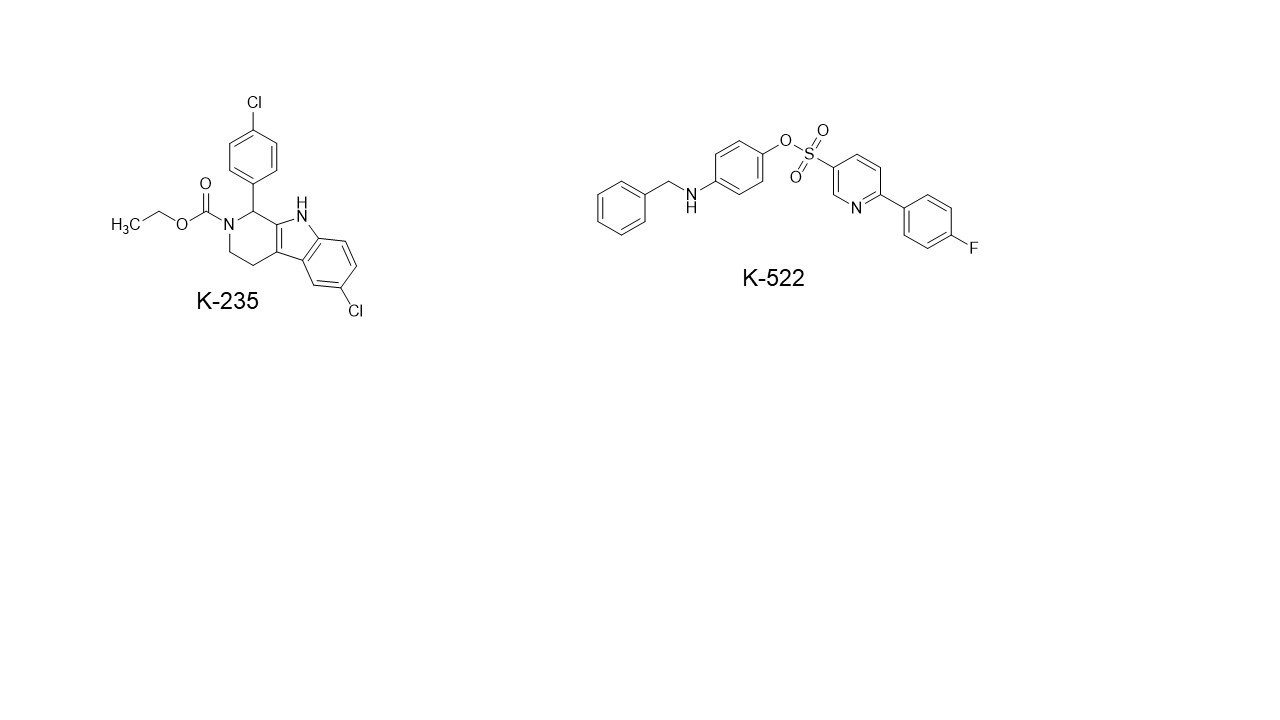


Figure S4. K-235 and K-522 influence the growth of Ewing’s sarcoma.

**A**, Ewing’s sarcoma-derived TC-71 cells and human pancreas adenocarcinoma-derived AsPC-1 cells as a control were treated with indicated doses of K-235 for 72 h, and cell viability was measured. Each value represents the mean of duplicate measurements. **B,C,** Ewing’s sarcoma with type A-fusion gene derived A-673, type 2-fusion gene-derived SK-ES-1, RD-ES, and MHH-ES-1 cells and Ewing’s sarcoma with EWS-ERG fusion gene-derived CADO-ES-1 cells were treated with indicated doses of K-235 or K-522 for 120 h, and cell viability was measured. Each value represents the mean of duplicate measurements. **D,** Chemical structures of K-235 and K-522


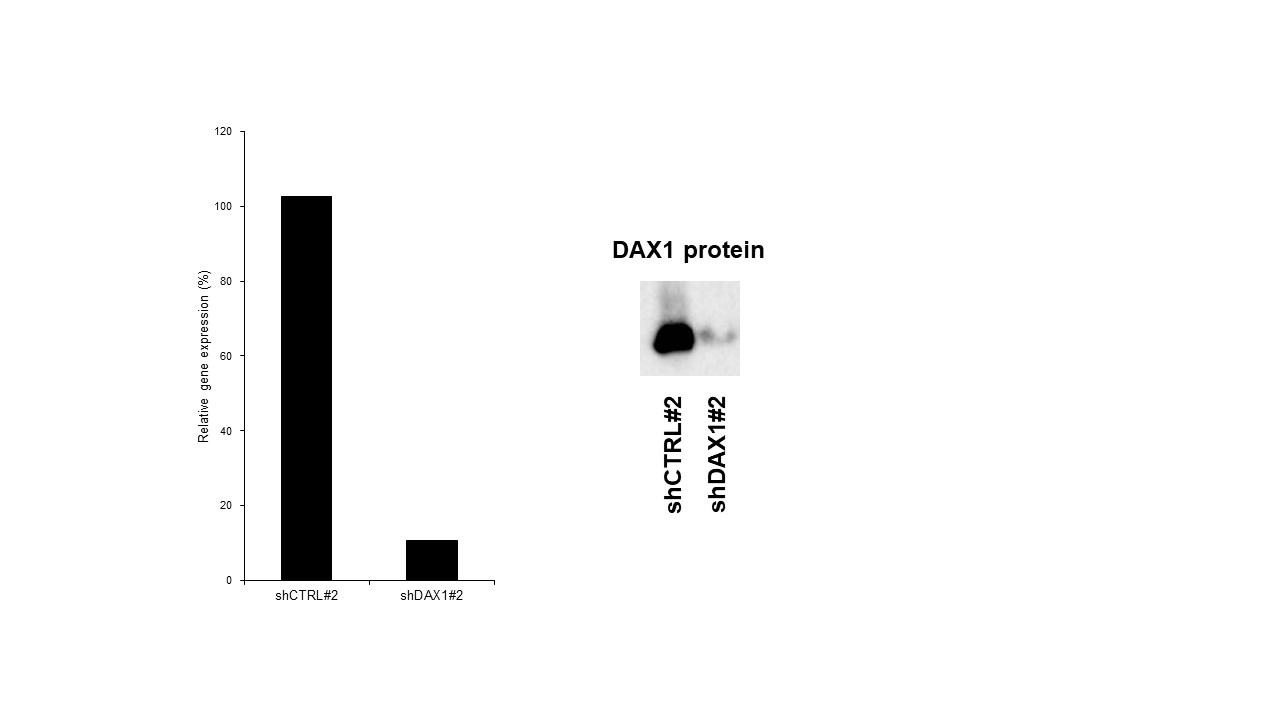


**Figure S5. DAX1 mRNA and protein levels after shDAX1 treatment.**

A-673 cells were infected with DAX1 (shDAX1#2) or control (shCTRL#2) shRNA-expressing lentivirus. *DAX1* expression was measured by RT-qPCR and normalized by GAPDH expression. DAX1 expression was measured by Western blotting.

**Materials & Methods S1**

*Plasmid construction*

The full-length cDNA of human DAX1 (NM_000475.4) was subcloned into the pcDNA3.1 expression vector (Thermo Fisher Scientific, Waltham, MA, USA) (pcDNA3.1-hDAX1) and transferred to a pAGal9 plasmid^a^ to obtain a human DAX1 expression plasmid (pAGal9p4-hDAX1). DNA with 10 copies of the PPARγ-response element (PPRE) derived from rat acyl-CoA oxidase gene^b^ with a TATA sequence (Fig. S1) and its antisense DNA was synthesized (Sigma-Aldrich, St. Louis, MO, USA), annealed, and subcloned into a pACREplucGI plasmid (pAPPRE×10-Luc+). The full-length cDNA of human DHODH (NM_001361.4) was subcloned into the pcDNA3.1 hygro(+) (Thermo Fisher Scientific) (pcDNA3.1hygro(+)-hDHODH).

^a^Saeki S, Kunitomo H, Narita Y, et al. A reporter assay for G-protein-coupled receptors using a B-cell line suitable for stable episomal expression. Analytical Biochem. 2010;400:163-172. doi:10.1016/j.ab.2010.01.036

^b^Sakamoto J, Kimnura H, Moriyama S, et al. Activation of Human Peroxisome Proliferator-Activated Receptor (PPAR) Subtypes by Pioglitazone. Biochem and Biophys Res Commun. 2000; 278:704-711. doi:10.1006/bbrc.2000.3868

Materials & Methods S2

*Cell culture*

A-673 cells were cultured in Dulbecco’s Modified Eagle Medium (DMEM; Thermo Fisher Scientific) with 10% fetal bovine serum (FBS; Thermo Fisher Scientific). TC-71 cells were grown in Iscove’s Modified Dulbecco’s Medium (IMDM; Thermo Fisher Scientific) with 10% FBS, and 1% penicillin-streptomycin (Nacalai Tesque, Kyoto, Japan). SK-ES-1 cells were cultured in McCOY’s 5A (Thermo Fisher Scientific) with 15% FBS and 1% penicillin-streptomycin. AsPC-1 and CADO-ES1 cells were cultured in RPMI1640 (Thermo Fisher Scientific) with 10% FBS and 1% penicillin-streptomycin. All cells were grown at 37 °C in 5% CO_2_/95% air. KJMGER8 is a Namalwa KJM-1 cell line stably expressing the GAL4–ER in the presence of 2 μg/ml blasticidin S, and Namalwa KJM-1 cells are a subline of the human Burkitt lymphoma Namalwa cell line^a^. KJMGER8 cells were cultured in RPMI1640-ITPSG medium containing 1 μg/mL puromycin, 0.3 μg/mL hygromycin, and 2 μg/mL blasticidin S. RPMI1640-ITPSG medium was prepared by adding 0.188% NaHCO_3_, 6 mM L-glutamine, 10 mM HEPES, 3 mg/L insulin, 5 mg/L transferrin, 5 mM sodium pyruvate, 125 nM sodium selenite, 1 mg/mL galactose, and 100 U/mL penicillin-streptomycin to RPMI1640^a^.

^a^Saeki S, Kunitomo H, Narita Y, et al. A reporter assay for G-protein-coupled receptors using a B-cell line suitable for stable episomal expression. Analytical Biochem. 2010;400:163-172. doi:10.1016/j.ab.2010.01.036

Materials & Methods S3

*Reporter gene assays*

pAGal9-hDAX1 and pAPPRE×10-Luc+ were transfected into KJMGER8 cells by electroporation using a Gene Pulser (Bio-Rad Laboratories, Hercules, CA, USA), and clones stably expressing the DAX1 and luciferase reporter genes under the control of PPRE were selected. The obtained clone was termed KJMGER8/pAGal9p4-hDAX1. KJMGER8/pAGal9p4-hDAX1 cells (1.8×10^4^ cells/well) were seeded in 384-well white plates (Greiner Japan, Tokyo, Japan) and cultured in RPMI1640-IPTSG medium containing 10 nM 17β-estradiol (E2; Sigma-Aldrich), dissolved in ethanol, with test compounds at concentration ranges of 0.01 to 10000 nM or vehicle (dimethyl sulfoxide [DMSO]). The final DMSO concentration was ≤0.3%, and the ethanol concentration was 0.1%. The next day, Blight-Glo Luciferase Assay Reagent (Promega, Madison, WI, USA) was added to the medium. The luciferase activity was measured using a TopCount NXT system (PerkinElmer, Waltham, MA, USA). IC_50_ values were calculated using XLfit version 4 (ID Business Solutions, Surrey, UK)

Materials & Methods S4

*RNA interference and lipofection*

RNA interference: small hairpin RNA (shRNA) targeting human DAX1 (5’-TGCAGTGCGTGAAGTACATTC-3’ and 5’-TGCGCTTCGTCAAGTACTTGC-3’) and small interfering RNA (siRNA) targeting human FLI1 (5’-CGATCAGTAAGAATACAG-3’) were purchased from Sigma-Aldrich. shDAX1 was inserted into pLKO.1 (Sigma-Aldrich‎). The plasmid was infected to A-673 cells and cultured overnight, followed by selection with 0.25 µg/mL puromycin in DMEM. siFLI1 was transfected into A-673 cells using Lipofectamine RNAiMAX transfection reagent (Thermo Fisher Scientific), and the cells were cultured in OPTI-MEM (Sigma-Aldrich). Twenty-four hours later, the medium was removed and replaced with DMEM.

Lipofection: pcDNA3.1-hDAX1 or pcDNA3.1hygro-hDHODH was transfected to A-673 cells using TransIT-LT1 reagent according to the manufacturer’s protocol. These A-673 cells were subjected to hygromycin antibiotic selection for 6 days (hygromycin concentration, 700 µg/mL).

Materials & Methods S5

*Quantitative reverse transcription polymerase chain reaction (RT-qPCR)*

Total RNA was extracted using the RNeasy Plus Mini kit (Qiagen, Valencia, CA, USA) according to the manufacturer’s protocol. cDNA was synthesized from 1 μg of total RNA using a SuperScript VILO cDNA synthesis kit (Thermo Fisher Scientific). Real-time PCR was carried out using TaqMan Fast Universal PCR Master Mix (Thermo Fisher Scientific). The primers used are listed in Table S1.

Materials & Methods S6

*Pharmacokinetic analyses*

Male BALB/cAnNCrlCrlj mice (6 weeks old) were purchased from Jackson Laboratories Japan (Yokohama, Japan). K-733, suspended in 0.5% (w/v) MC400 was orally administered at 1, 3, 10, and 100 mg/kg (n=2), and blood samples were collected from the tail vein at 0.5, 1, 2, 4, 7 and 24 h after administration. Mice were euthanized with carbon dioxide inhalation after the final blood sampling. Plasma was separated and stored frozen until use. The plasma concentration of K-733 was determined by liquid chromatography with tandem mass spectrometry (LC-MS). Plasma samples were pre-treated for protein precipitation, and the supernatant was subjected to liquid LC-MS. The analyte was separated on a C18 reverse-phase column (CAPCELL PAK C18 MG 3 μm, 3 mm I.D. × 35 mm; Osaka Soda). LC-MS detection was performed by electrospray ionization in a positive ion mode. The detected mass-to-charge ratio of K-733 was 482.95/92.75 (Q1/Q3). The lower limit of quantification (LLOQ) was 10 nM.
